# Decoding interaction between mitochondria and endoplasmic reticulum in ischemic myocardial injury: targeting natural medicines

**DOI:** 10.3389/fphar.2025.1536773

**Published:** 2025-02-28

**Authors:** Chuxin Zhang, Xing Chang, Dandan Zhao, Yu He, Guangtong Dong, Lin Gao

**Affiliations:** ^1^ School of Traditional Chinese Medicine, Beijing University of Chinese Medicine, Beijing, China; ^2^ Guang’anmen Hospital of Chinese Academy of Traditional Chinese Medicine, Beijing, China

**Keywords:** ischemic cardiomyopathy, inflammatory vascular injury, mitochondrial quality control, endoplasmic reticulum, oxidative stress, traditional chinese medicine

## Abstract

Ischemic cardiomyopathy (ICM) is a special type or end stage of coronary heart disease or other irreversible ischemic myocardial injury. Inflammatory damage to coronary vessels is a crucial factor in causing stenosis or occlusion of coronary arteries, resulting in myocardial ischemia and hypoxia, but it is also an aspect of cardioprotection that is often overlooked. This review discusses the mechanisms of vascular injury during ICM, in which inflammation and oxidative stress interact and trigger cell death as the cause of coronary microvascular injury. Imbalances in endoplasmic reticulum function and mitochondrial quality control are important potential drivers of inflammation and oxidative stress. In addition, many studies have confirmed the therapeutic effects of Chinese herbal medicines and their natural monomeric components on vascular injuries. Their mitochondrial quality control and endoplasmic reticulum protection mechanisms as well as their role in combating improvements in vascular endothelial function and attenuating vascular injury are also summarized, with a perspective to provide a reference for pathologic understanding, drug research, and clinical application of ICM-associated coronary microvascular injury.

## 1 Introduction

Ischemic cardiomyopathy (ICM) is a specific type or end stage of coronary heart disease or other irreversible ischemic myocardial injury. It is mainly due to coronary atherosclerosis caused by long-term coronary artery stenosis or occlusion, resulting in long-term myocardial ischemia and hypoxia, irreversible damage to myocardial cells and irreversible cardiac remodelling (Chang et al., 2023a; Pastena et al., 2023). A series of clinical symptoms, such as angina pectoris, arrhythmia, heart failure, thrombosis and embolism follow (Panza et al., 2019), seriously threatening people’s physical and mental health (Khan et al., 2020; Virani et al., 2021).

**FIGURE 1:**
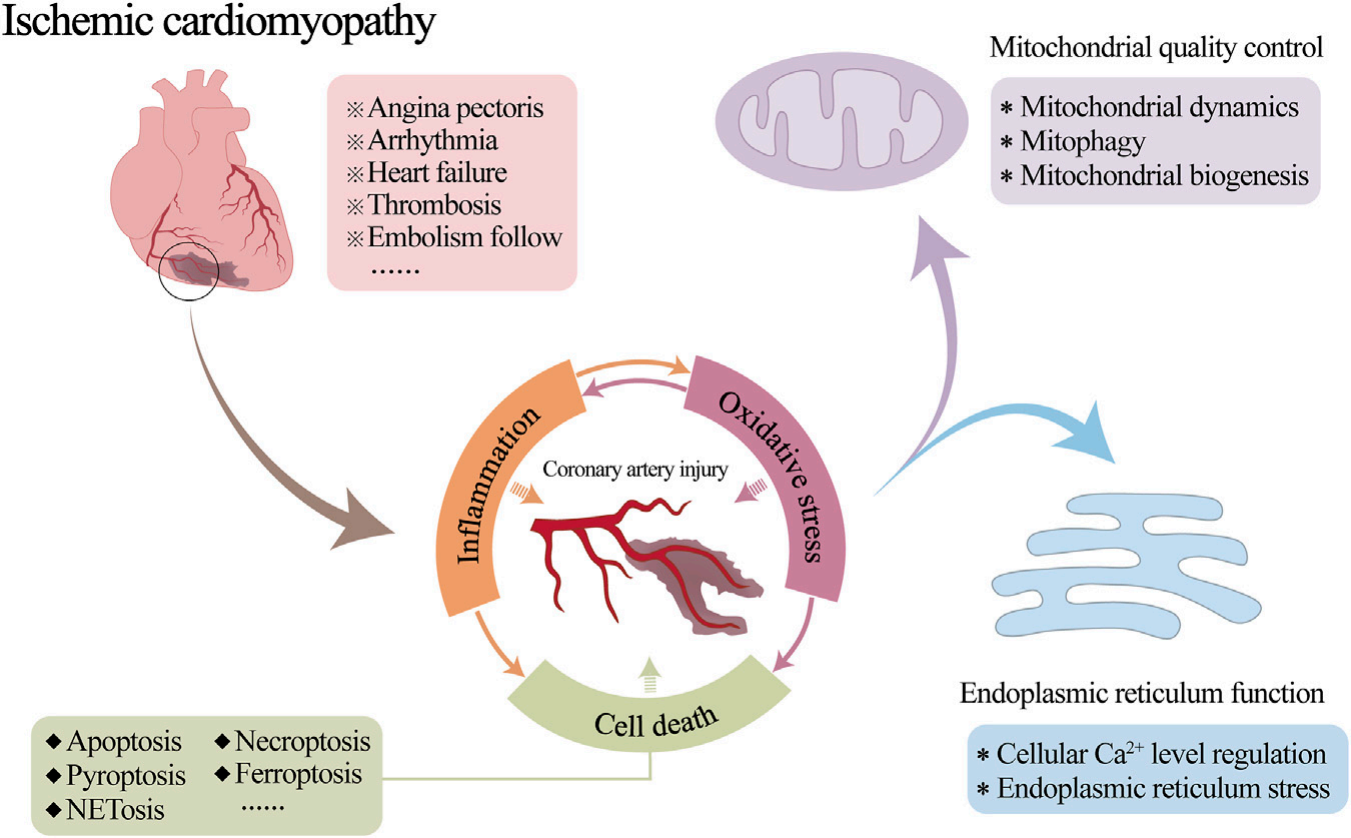
Pathological mechanism of coronary artery injury in ischemic cardiomyopathy (ICM).

Coronary blood flow is essential for the normal physiological function of the heart. Therefore, reduced local blood flow due to coronary artery stenosis or occlusion is often a key cause of the development of myocardial ischemia (Efentakis et al., 2024; Li S. Y. et al., 2024). Inflammatory damage to the coronary vasculature is one of the primary pathological changes in this process (Chang et al., 2023b; Zhang X. M. et al., 2023). Although the medical community has some understanding of the mechanism by which mitochondrial and endoplasmic reticulum (ER) abnormalities are involved in the occurrence and development of myocardial injury after myocardial infarction or ischemia-reperfusion (I/R), coronary circulation is still a neglected cardiac protection target so far. Its pathological mechanism in the process of ICM is not yet clear. The relevant targeted therapeutic drugs need to be further studied. A large number of studies have shown that mitochondrial quality control (MQC) and ER regulatory mechanisms are involved in regulating the whole process of vascular physiological and pathological functions (Tan et al., 2020; Hou D. et al., 2024; Li J. et al., 2024). However, in the development of ICM, the effects of both on vascular inflammatory injury are still unclear. Consequently, it is necessary to further explore the interaction mechanism of MQC and ER function involved in vascular physiology and pathology to directly link the ICM with ICM-related vascular injury (Figure 1).

Traditional Chinese medicine (TCM) has a long history of use in China, with rich drug resources and a unique theoretical system. In recent years, the research and clinical application of the main active monomers of TCM have also increased its vitality and value. Over the past two decades, many studies have demonstrated that TCM compounds, drug extracts, and related monomer drugs are able to reduce oxidative stress, inflammatory responses, and cell death by modulating MQC, ER function, and counteracting vascular injury. However, few studies have investigated the efficacy and mechanism of action of natural drugs for coronary microvascular injury during the development of ICM, which has great research value and clinical application prospects.

This article outlines the current understanding of ICM-associated inflammatory vascular injury, the interaction between oxidative stress and inflammation, and inflammation-mediated programmed cell death. The critical role of ER function and the MQC system in vascular pathology is a key focus. Furthermore, this article summarizes the vascular targeting effects of natural drugs and their monomer components that have been reported so far, with a perspective to provide a reference for drug research and clinical application in ICM-related coronary microvascular injury.

## 2 Pathologic mechanisms of inflammatory vascular injury

### 2.1 Inflammatory damage

The inflammatory process of ICM involves a variety of pathological mechanisms. Among them, oxidized LDL (ox-LDL) (Ammar et al., 2022), advanced glycation end products (AGEs) (Zhang J. et al., 2023), stable activation of hypoxia-inducible factor 1α (HIF-1α) caused by hypoxia (Du et al., 2018), and destruction of the endothelial glycocalyx barrier (Dehghani et al., 2022) are important pathological nodes for activating and promoting the vascular endothelial inflammatory response.

Endothelial cells (ECs) play a key role in maintaining vascular homeostasis by regulating vascular tone, permeability, and angiogenesis and are targets for anti-inflammatory and antithrombotic factors. It is both the target of inflammatory stimulation and the main promoter of inflammation. Under conditions of hypoxia, hyperlipidemia, hyperglycemia, oxidative stress and other adverse factors, ECs express vascular cell adhesion molecule-1 (VCAM-1), intercellular adhesion molecule-1 (ICAM-1), interleukin-6 (IL-6), IL-1, monocyte chemoattractant protein-1 (MCP-1), and other inflammatory cytokines (Schunk et al., 2021; Wang et al., 2021; Takenoshita et al., 2024). These inflammatory factors stimulate the activation, adhesion and infiltration of circulating leukocytes (including neutrophils, lymphocytes, and monocytes) (Tang et al., 2023), resulting in vascular endothelial injury. Damaged tissue further releases proinflammatory cytokines, resulting in reactivation of the proinflammatory signalling pathway and the enhancement of the inflammatory response (Zhao F. et al., 2023). In this process, toll-like receptor 4 (TLR4) and its downstream transcription factor nuclear factor kappa-B (NF-κB) perform essential functions. Adequate activation of NF-κB is necessary for the expression of major proinflammatory mediators such as tumor necrosis factor-α (TNF-α), IL-1, and IL-6 (Li et al., 2023b). TLR4, a typical pattern recognition receptor, is preferentially expressed on the surface of vascular ECs. When activated by MyD88-targeted NF-κB, TLR4 triggers the occurrence and cascade enhancement of the inflammatory response (Kiyan et al., 2019; Liu et al., 2021). Excessive inflammatory stimulation eventually leads to impaired vascular tone, increased permeability, increased procoagulant activity, and dysregulated angiogenesis (Zhao H. et al., 2023), worsening the progression of ICM.

### 2.2 Oxidative stress and inflammation

Oxidative stress refers to the process in which excessive reactive oxygen species (ROS), such as superoxide (O_2_^−^), peroxy radical (ROO^−^), and hydrogen peroxide (H_2_O_2_), are produced by the system under various pathological stimuli, which are strongly unbalanced with the antioxidant capacity of the biological system, causing toxic reactions leading to cell death and tissue damage (Ramachandra et al., 2020). During the ICM, mitochondrial reverse electron transfer (RET) and nicotinamide adenine dinucleotide phosphate (NADPH) oxidase (NOX) family enzymes are the main sources of ROS in the coronary microvascular (Manuneedhi Cholan et al., 2018; Feenstra et al., 2023).

There is a close relationship between oxidative stress and the inflammatory response. Through interaction, the two can promote the progression of a series of pathological processes of the ICM, such as abnormal vasoconstriction, fibrosis, and thrombosis (D’Onofrio et al., 2023; Kulkovienė et al., 2024). Recent studies have shown that TNF-α-treated human aortic endothelial cells (HAECs), coronary artery endothelial cells (HCAECs), and umbilical vein endothelial cells (HUVECs) all display activation of inflammatory pathways, mitochondrial abnormalities, and ROS accumulation (D’Onofrio et al., 2023). The inhibition of NF-κB p65 reduces ROS production in ox-LDL-stimulated HUVECs (Li Y. et al., 2021). In addition, neutrophils are one of the sources of ROS. ROS from ECs can amplify the inflammatory response and affect nearby neutrophils, further inducing a cascade of ROS generation (Nakamura et al., 1998; Hori and Nishida, 2008). At the same time, the increase in ROS production in inflammatory tissues during the inflammatory process significantly promotes the synthesis of proinflammatory mediators such as cytokines and chemokines involved in inflammatory cell migration (Shukla et al., 2020; Tan et al., 2022). Mitochondrial DNA (mtDNA) is oxidized and destroyed under oxidative stress, which escapes into the cytoplasm through the mitochondrial permeability transition pore (mPTP) and causes an inflammatory response (Xian et al., 2022; An et al., 2023). Excess ROS can also cause low-density lipoprotein (LDL) to peroxidize into ox-LDL (Olivares-Caro et al., 2020). Ox-LDL can upregulate the expression of p-selectin, VCAM, ICAM and MCP-1 on the cell surface, accelerate the adhesion and penetration of white blood cells to the vascular endothelium (Lei et al., 2008; Ammar et al., 2022; Luo et al., 2024), and lead to an enhanced inflammatory response. Studies have shown that by activating NF-κB, ROS can enhance the expression of the inflammatory cytokines ICAM-1, VCAM-1, IL-6 and TNF-α in vascular endothelial tissue (Kim et al., 2008; Han et al., 2015), and increase the adhesion characteristics of ECs (Alshabibi et al., 2018). In summary, there is a complex interaction between oxidative stress and inflammation, which is a key factor in promoting vascular injury and ICM progression.

### 2.3 Inflammation-mediated programmed cell death

In the ICM, the activation and amplification of the inflammatory response leads to programmed cell death of vascular ECs and inflammatory cells (such as macrophages and neutrophils), which involves mainly apoptosis, necroptosis, pyroptosis, ferroptosis and NETosis. Under oxidative stress, mitochondria produce excessive ROS and determine cell fate, which is the central link between inflammation-mediated apoptosis, necroptosis, pyroptosis, ferroptosis and other cell death pathways (Basit et al., 2017). Together, these processes lead to vascular tissue damage, plaque calcification, lesion thrombosis, and coronary artery stenosis (Zhang A. et al., 2023; Jiang et al., 2024; Lu et al., 2024), exacerbating the progression of ICM.

In coronary artery ECs, inflammation-mediated apoptosis is generally considered to be an indirect effect, that is, by inducing the explosive production of ROS, causing the destruction of mitochondrial membrane components, activating Bax, inducing the increase of mitochondrial membrane permeability and cytochrome C (cyt C) release (Salie et al., 2023). Cyt C binds to the C-terminal domain of apoptotic protease activating factor-1 (Apaf-1), induces conformational changes, activates the release of caspase-9 and caspase-3, and ultimately accelerates the apoptosis of ECs (Miao et al., 2018; Xue et al., 2023).

Necroptosis is programmed cell death induced by a class of death receptors (TNFR1, Fas and TRAIL-R). The inflammatory factor TNF-α can initiate necroptosis through TNFR1 (Nam et al., 2024). By binding to the extracellular part of TNFR1, TNF-α causes allosteric changes in the intracellular part of TNFR1 (Huang et al., 2022) and mediates the activation of downstream molecules such as receptor-interacting serine threonine kinase 1 (RIPK1), RIPK3, Fas-associated with death domain protein (FADD)/TNF receptor-associated death domain protein (TRADD), and caspases 8/10 (Xiao et al., 2020). Subsequently, RIPK1, RIPK3, and mixed lineage kinase domain like protein (MLKL) form a necrotic complex, which promotes the opening of the mitochondrial mPTP (Szobi et al., 2018; Qiang et al., 2022; Li Z. et al., 2023), mediates mitochondrial damage, and ultimately induces cell death (Pozzer et al., 2019). In addition, recent studies have shown that RIPK1 can drive NF-κB-dependent inflammation in early atherosclerotic lesions and mediate the initiation of necrotic apoptosis in macrophages and ECs (Karunakaran et al., 2021). At the same time, the expression of RIPK1 is also a key link in the process of TNF-induced necroptosis (Newton et al., 2019).

Pyroptosis is a form of cell death that is dependent on caspase-1 activation and is widely involved in the occurrence, progression and complications of ICM (Jin et al., 2023). It is characterized by rapid rupture of the plasma membrane and the release of proinflammatory substances in cells. Recent studies have shown that the classical pathway of pyroptosis is mediated by the NLRP3 inflammasome in ICM vascular injury (Shao et al., 2022; Li S. et al., 2024; Yang et al., 2024). The NLRP3 inflammasome is a macromolecular protein complex, and its three key components include the NLRP3 protein, apoptosis-associated speck-like protein containing a CARD (ASC), and procaspase-1. It can sense damage by activating caspase-1, and then stimulate the release of proinflammatory cytokines IL-1β and IL-18 and the cleavage of Gasdermin D (GSDMD), triggering and amplifying the inflammatory response. This process is one of the drivers of pyroptosis (Toldo and Abbate, 2024). Mitochondrial damage and dysfunction will increase the production of ROS and ox-LDL and K^+^ efflux (Jiang et al., 2023; Kan et al., 2024; Tao et al., 2024), which trigger the inflammatory response of NLRP3, induce pyroptosis of inflammatory cells and ECs, and aggravate vascular endothelial damage.

Ferroptosis is a new type of programmed cell death that has attracted wide attention in recent years. Excessive accumulation of lipid peroxides leads to cell death in the case of iron-dependent overload. Iron metabolism disorders, oxidative stress, lipid peroxidation, targeted induction of P53, and other factors are involved in the occurrence of ferroptosis. Studies have shown that TNF-α may be involved in the process of promoting ferroptosis in vascular ECs (Chen X. et al., 2023; Jankauskas et al., 2023). In addition, ferroptosis has been shown to be involved in the drive of proinflammatory response (Hou H. et al., 2024). Iron overload in the arterial wall results in increased expression of cytokines, increased expression of ICAM-1 and VCAM-1, extensive endothelial defects and increased permeability, and excessive oxidative damage (Vinchi et al., 2020). Ferroptosis inhibitors can reduce subsequent immune cell infiltration and the inflammatory response (Li et al., 2019; Zhu et al., 2024), which may have a timely preventive effect on the extensive vascular injury caused by inflammation.

NETosis is a neutrophil-specific form of programmed cell death involving the release of neutrophil extracellular traps (NETs) (MacArthur et al., 2024). NETs are reticular structures composed of DNA, histones and various antimicrobial proteins. They are released by neutrophils to capture and destroy extracellular pathogens such as bacteria and fungi. During NETosis, neutrophils undergo a series of morphological changes, leading to cell membrane rupture, and NETs are released into the surrounding environment, inducing the activation of ECs, antigen-presenting cells and platelets, leading to local tissue inflammation and promoting vascular injury and thrombosis (Sun et al., 2023). Moreover, dysfunctional ECs will enhance NETosis, amplify the damage to adjacent cells, and further damage the inner layer of the arterial endothelium (Nija et al., 2020). Peptide arginine deiminase 4 (PAD4) is a key enzyme in NETosis, which mediates the citrullination of histones and the decondensation of chromatin, resulting in DNA extrusion and NETs formation. Studies have shown that in the Ldlr^−/−^ mouse model of blood flow-mediated superficial intimal erosion, the inactivation of the PAD4 gene can eliminate NETosis, reduce endothelial permeability, and reduce *in situ* thrombosis (Dou et al., 2021). In addition, a decrease in TUNEL^+^ apoptotic ECs was observed in mice treated with Pad4^−/−^ bone marrow transplantation or PAD4 inhibitors (Franck et al., 2018). Recent studies have also shown that targeted delivery of PAD4 inhibitors in ApoE^−/−^ mouse models can reduce the accumulation of NETs at the site of intimal injury and maintain endothelial continuity (Molinaro et al., 2021).

## 3 ER interacts with vascular physiology and pathology

### 3.1 ER physiological regulatory mechanism

The ER is a multifunctional organelle that plays an important role in a variety of physiological processes, which is involved in protein synthesis, folding and translocation, and regulates cellular Ca^2+^ uptake, storage and signalling. In addition, the ER is involved in the production of cellular lipids, such as cholesterol, glycerophospholipids, and ceramides, as well as the regulation of gene expression and energy metabolism, and the transmission of signals to the nucleus, cytoplasm, mitochondria, and plasma membrane (Dong et al., 2024).

**FIGURE 2:**
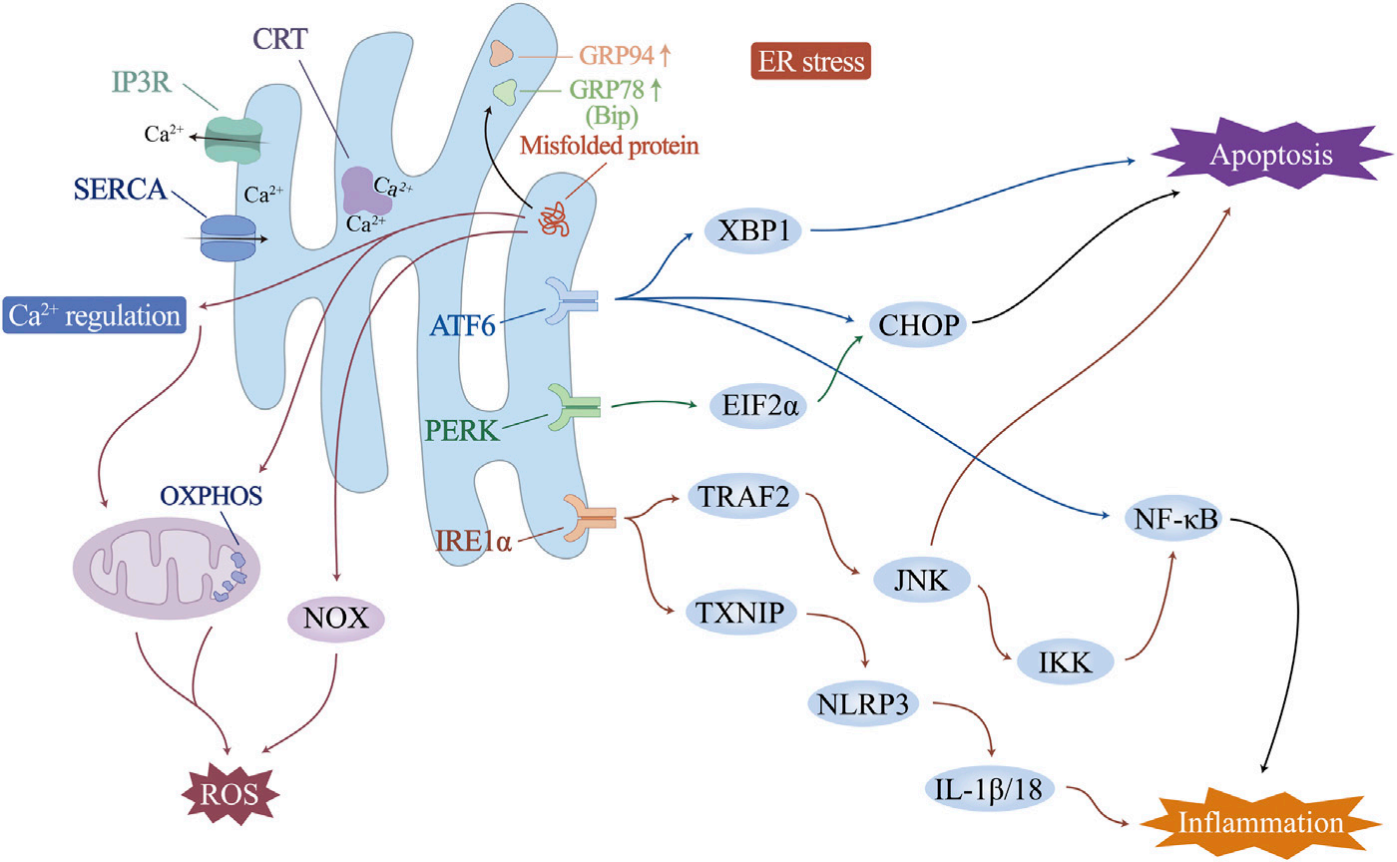
Endoplasmic reticulum (ER) Ca^2+^ regulation and ER stress.

Increased physiological and pathological changes, including fluctuations in intracellular Ca^2+^ levels, genetic or environmental damage, oxidative stress, inflammatory responses, and glycosylation, may disturb the steady state of the ER and impair its ability to fold and posttranslationally modify proteins. When the load of secreted proteins exceeds the folding capacity of the endoplasmic reticulum, it leads to the accumulation of misfolded proteins, which is called endoplasmic reticulum stress (ERS). In this case, transcription-induced endoplasmic reticulum chaperone genes such as glucose-regulated protein 78 (GRP78)/binding protein (BiP) or glucose-regulated protein 94 (GRP94) are activated to promote folding ability. ER-associated protein degradation pathway (ERAD) and the unfolded protein response (UPR) are activated to accelerate the degradation of unfolded proteins. Endoplasmic reticulum remodelling and ER autophagy will be initiated to remove excess ER membranes and proteins, fight ERS, and maintain normal ER structure and function (Figure 2).

### 3.2 ER is involved in regulating vascular endothelial physiology

The vascular endothelium is a unique, dynamically regulated organ composed of monolayer ECs that regulate blood flow and fibrosis, vascular tone, angiogenesis, vascular permeability, leukocyte adhesion, and platelet aggregation. It is essential for maintaining vascular homeostasis and adapting the cardiovascular system to environmental changes. Ca^2+^ is a ubiquitous secondary messenger that initiates signal transduction events in ECs. It regulates the synthesis and release of various vasoactive substances, such as nitric oxide (NO), prostacyclin (PGI2), endothelium-derived hyperpolarizing factor (EDHF), and endothelin, so that ECs can achieve normal physiological functions such as the regulation of vascular tension, endothelial permeability, and angiogenesis. As one of the intracellular organelles that regulate the uptake and storage of Ca^2+^, the ER plays an important role in the physiological regulation of the vascular endothelium (Suzuki et al., 2014). The ER in vascular ECs mainly controls Ca^2+^ efflux through inositol triphosphate receptor (IP3R) and Ca^2+^ influx through sarco/endoplasmic reticulum Ca^2+^-ATPase (SERCA) (Cohen and Jackson, 2005; Yan et al., 2015). Studies have shown that IP3R is involved in the negative feedback regulation of small artery muscle tone by regulating endothelial cell Ca^2+^ levels (Cohen and Jackson, 2005; Ledoux et al., 2008). Under the regulation of NO, SERCA can reduce the intracellular free Ca^2+^ by increasing the uptake of Ca^2+^ and play the role of reducing vascular tension (Adachi et al., 2002; Adachi et al., 2004). SERCA overexpression has been shown to have protective effects on endothelial function and barrier integrity in many studies (Li et al., 2020a; Tan et al., 2020). In addition, a Ca^2+^-binding protein with high affinity and Ca^2+^ buffering capacity, calreticulin (CRT), is present in the ER and plays a supportive role in adequate ER Ca^2+^ storage and stable Ca^2+^ levels (Wang et al., 2018; Wang et al., 2019b).

### 3.3 ER is involved in the mechanism of vascular endothelial pathological injury

The ER is involved mainly in vascular endothelial pathological damage through Ca^2+^ homeostasis and ERS.

Adverse factors such as high glucose, high fat, ischemia and hypoxia, and inflammatory infiltration that may exist in the pathological state of the ICM can stimulate the abnormal expression of IP3R and SERCA in the ER, causing a rapid increase in intracellular Ca^2+^ levels (Qin et al., 2014; Zhang et al., 2016; Yu et al., 2017; Zhou et al., 2018; Chen et al., 2021). This leads to Ca^2+^ -dependent xanthine oxidase (XO) activation and ROS production, which in turn triggers endothelial cell apoptosis (Liu M. et al., 2024). SERCA overexpression can reduce cardiac microvascular endothelial cell death and exert a protective effect against cardiac microvascular ischemia/reperfusion injury by improving mitochondrial quality control and inhibiting the expression of mitochondrial calcium-monotransporter protein (MCU) (Li et al., 2020a; Tan et al., 2020). In addition, inhibition of IP3R expression reduces microvascular damage caused by Ca^2+^ overload and related oxidative stress (Zhu et al., 2018).

At the same time, the above adverse factors and Ca^2+^ homeostasis itself are the reasons for the continuous accumulation of misfolded proteins. When the accumulation of misfolded proteins in the lumen of the ER is excessive and beyond the normal physiological regulation, the ERS and the persistence of the UPR have an impact on the cells and radicalize the pathological process.

In ICM vascular endothelial injury, ERS and UPR are involved in mediating the inflammatory response, oxidative stress and cell death (Wu L. et al., 2014; Huang et al., 2018; Wang F. et al., 2019). In particular, the UPR is controlled by three ER transmembrane proteins: protein kinase R-like endoplasmic reticulum kinase (PERK), inositol-requiring enzyme 1α (IRE1α), and activating transcription factor 6 (ATF6). Endoplasmic reticulum stress and subsequent endothelial cell apoptosis, involving activation of eukaryotic initiation factor 2α (EIF2α), X-box-binding protein 1 (XBP1), CCAAT-enhancer-binding protein homologous protein (CHOP), and NF-κB, can induce endothelial dysfunction. Correspondingly, ERS inhibition improves endothelial cell function directly or indirectly (Zhou et al., 2023). Upon UPR activation, IRE1α elevates the expression of TNF receptor-associated factor 2 (TRAF2), which, together with JNK and IκB kinases, initiates NF-κB and activates the expression of inflammatory cytokines (Lei et al., 2023). The ATF6 pathway has also been shown to trigger the NF-κB pathway (Nakajima et al., 2011; Chen et al., 2018). The IRE1 pathway induces an increase in thioredoxin-interacting protein (TXNIP), activates NLRP3 inflammatory vesicles, and promotes secretion of IL-1β and IL-18 (Hong et al., 2021). In addition, sustained ERS drives macrophage polarization to proinflammatory M1 morphology, causing organ inflammation during obesity and insulin resistance (Shan et al., 2017). Studies have shown that excess ER cholesterol triggers the macrophage UPR and activates the IκB/NF-κB, MAP kinase 3 (MKK3)/p38, extracellular regulated protein kinases (Erk) 1/2, and JNK1/2 signalling pathways, leading to elevated expression of the inflammatory molecules IL-8, IL-6, MCP-1 and TNF-α. This may be one of the mechanisms that exacerbates the vascular inflammatory response and macrophage accumulation in advanced atherosclerosis and ICM (Li et al., 2005).

ERS can activate ROS production in several ways: i) Activating of NOX, especially NOX2 and NOX4 (Lee et al., 2014; Vilas-Boas et al., 2021). ii) Ca^2+^ leaks from the ER lumen into the cytosol, which can increase mitochondrial ROS production (Zhang et al., 2016). iii) Increased energy consumption by the ER during protein folding and refolding stimulates mitochondrial oxidative phosphorylation to increase ATP and reactive oxygen species production, especially O_2_^−^. O_2_^−^ can combine with NO synthesized by endothelial NO synthase (eNOS) to form peroxynitrite, leading to impaired endothelium-dependent vasodilatation and decreased NO bioavailability, which in turn leads to endothelial dysfunction (Cheng et al., 2021; Hassoun et al., 2021). Conversely, oxidative stress may similarly promote ERS directly or indirectly. Excess mitochondrial ROS promote ER Ca^2+^ release and activate ERS (Kim et al., 2016). In addition, ox-LDL, a product of ROS-promoted lipid peroxidation formation, attenuates cholesterol efflux from ECs, upregulates the expression of ERS-associated proteins (CHOP, p-PERK, GRP78, and p-IRE-1) (Hang et al., 2020), and mediates the onset of ERS, the eruption of an inflammatory response, and, ultimately, endothelial cell apoptosis (Li P. et al., 2021). Promoting SOD1 through activation of Nrf2, which in turn counteracts ROS production, effectively alleviates ERS-induced endothelial cell apoptosis (Ei et al., 2022). These studies all support the interaction and synergy between oxidative stress and ERS, with ROS being both a downstream product of ERS and a regulator of ERS.

High levels of chronic ERS induce apoptosis through all three branches of the UPR. IRE1α initiates apoptosis via TRAF2 (Ma et al., 2023). The IRE1α cytoplasmic structural domain recruits TRAF2, which activates apoptosis signal-regulated kinase 1 (ASK1)/cJUN nh2-terminal kinase (JNK) (Ji et al., 2020; Kang et al., 2024). By stimulating the proapoptotic protein Bax and inactivating the antiapoptotic protein Bcl-2, JNK plays a role in apoptosis. PERK/EIF2α and ATF6 activate an increase in the level of the proapoptotic transcription factor CHOP, which initiates apoptosis by downregulating the expression of Bcl-2 and activating the cascade of caspase-12/9/3 (Peng et al., 2022; Li H. et al., 2024). CHOP also stimulates endoplasmic reticulum Ca^2+^ release and triggers the calcium-sensitizing enzyme CaMKII, which induces a variety of downstream apoptotic mechanisms such as Fas death receptor, JNK activation, and the release of mitochondrial cytochrome c, triggering apoptosis (Dilshara et al., 2018; He et al., 2021; Mohan et al., 2023). In addition, it has also been recently shown that ERS is also involved in activating other forms of cell death. Under hypoxia induction, ERS may also be involved in the autophagy of ECs through the IRE1-mediated UPR (Tao et al., 2023). Cigarette tar mediates RIPK3-dependent necroptosis and accelerates the progression of atherosclerosis by activating the ERS PERK/EIF2α/CHOP axis in vascular smooth muscle cells (Bai et al., 2024).

## 4 Mitochondria interact with vascular pathology

**FIGURE 3:**
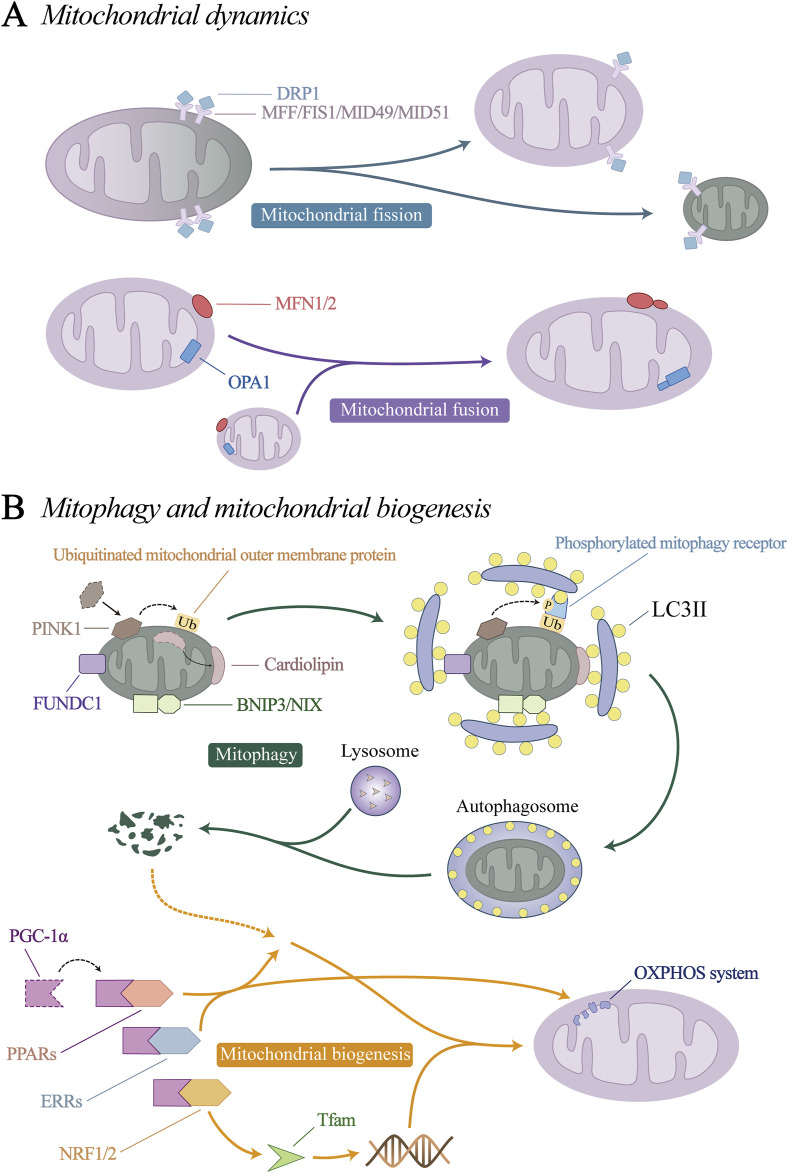
Mitochondrial quality control (MQC) mechanism. **(A)** Mitochondrial dynamics. **(B)** Mitophagy and mitochondrial biogenesis.

Mitochondrial quality control (MQC) is a group of adaptive responses that regulate mitochondrial dynamics, mitophagy, and mitochondrial biogenesis (Figure 3). It is an important mechanism to ensure mitochondrial homeostasis and maintain vascular endothelial stability (Liu et al., 2023; Ronayne et al., 2023; Tahmaz et al., 2023).

### 4.1 Mitochondrial dynamics and vascular pathology

Mitochondrial dynamics consists of mitochondrial fission and fusion processes. Through constant division and fusion, mitochondria maintain their integrity, distribution and size.

Mitochondrial fission can remove dysfunctional or damaged mitochondria from the mitochondrial network, which is important for maintaining the overall homeostasis and function of mitochondria (Suen et al., 2010; Kalkhoran et al., 2022). Mitochondrial fusion is a process of integrating several mitochondrial fragments into filamentous mitochondria, which can maintain the balance of mitochondrial components and the stability of function, weaken the damage to mtDNA and protein, and prevent the excessive division of mitochondria from causing apoptosis (Pirzeh et al., 2019; Gao et al., 2023; Noone et al., 2023). Mitochondrial dynamics not only determines mitochondrial homeostasis in many cell types, but also is considered to be a key requirement for vascular endothelial stability under normal conditions.

Mitochondrial fission is regulated by dynein-related protein 1 (DRP1) and its receptor mitochondrial fission factor (MFF), mitochondrial fission 1 (FIS1), mitochondrial dynamics protein 49 kDa (MID49) and MID51 (Ke et al., 2023; Ren et al., 2023). Under physiological conditions, DRP1 is mainly free in the cytoplasm in an inactive form, so mitochondrial fission is relatively rare. Under stress conditions, DRP1 undergoes conformational changes through posttranscriptional modification, such as ubiquitination, acetylation, and phosphorylation (Wang X. et al., 2019; Hu et al., 2023; Li et al., 2023c). Its binding sites are exposed and transported to the mitochondrial surface to bind to its receptors, where it mediates mitochondrial fission.

Mitochondrial fusion involves two processes. Mitofusin 1 and 2 (MFN1, MFN2) are located on the mitochondrial outer membrane (OMM) and mediate OMM fusion through homotypic or heterotypic coordination (Noone et al., 2023). Optic atrophy 1(OPA1) mediates endosomal fusion, which exists in two different forms, the long isomer of OPA1 (L-OPA1), and the short isomer (S-OPA1). Under the action of yeast mitochondrial escape 1 like 1 ATPase (YME1L1) and OMA1 zinc metallopeptidase (OMA1), L-OPA1 can be hydrolysed to S-OPA1 (Consolato et al., 2018; Jian et al., 2023). L- and S-OPA1 homeostasis coordinates to promote mitochondrial inner membrane fusion (Ge et al., 2021; Duan et al., 2023).

Studies have shown that mitochondrial dynamics are involved in mediating vascular pathology in the ICM, mainly through excessive mitochondrial fission, resulting in endothelial cell dysfunction and the development of vascular injury, degeneration, and fibrosis. *In vitro* studies have shown that DRP1 and FIS1 accumulate in HAECs exposed to high-glucose environments (Morales et al., 2014). In contrast, inhibition of DRP1 or FIS1 reduced mitochondrial fragmentation, attenuated mitochondria-derived ROS release, suppressed vascular inflammation and ameliorated endothelial dysfunction (Wang et al., 2017). MiD49/51 expression was elevated in aortic valve ECs from ApoE^−/−^ mice with high-fat diet-induced atherosclerosis, which accelerated DRP1-mediated mitochondrial fission. Silencing MiD49/51 reduces atherosclerotic plaque size and increases collagen content (Ren et al., 2023). In addition, animal studies provide evidence that acute microvascular I/R injury in mice is accompanied by increased MFF expression, excessive mitochondrial division, and mitochondria-dependent microvascular endothelial cell apoptosis. Compared with wild-type mice, MFF-deficient mice presented more stable coronary blood flow, less microcirculatory perfusion injury, a smaller infarct size, and preserved cardiac function during acute microvascular I/R injury (Zhou et al., 2017a). Clinical evidence suggests that mitochondrial breaks, mitochondrial ROS levels, and FIS1 expression are increased in isolated venous ECs from diabetic patients with one of the cardiovascular risk factors, hyperglycemic state, compared to healthy subjects, which may contribute to the induction of cardiovascular pathologic changes (Shenouda et al., 2011).

In contrast, mitochondrial fusion has been noted in studies to play a role in delaying vascular injury and ICM progression. The mitochondrial fusion proteins MFN1 and MFN2 maintain the mitochondrial network and protect endothelial cell function by initiating OMM fusion.Reduced expression of MFN1 and MFN2 leads to endothelial dysfunction and inhibition of VSMC proliferation, promoting the progression of atherosclerosis (Lugus et al., 2011; Forrester et al., 2020). Knockout of MFN2 impairs gene expression of mitochondrial respiratory chain and oxidative metabolism-related transcription factors, while knockout of MFN1 reduces Akt-mediated eNOS expression and inhibits endothelial cell activity (Lugus et al., 2011). Animal studies have shown that during cardiac microvascular I/R, the expression of MFN2 and OPA1 mRNA in mouse cardiac microcirculation vascular ECs was reduced, mitochondrial fusion was inhibited, vascular lumen was narrowed, vascular wall thickening, and the risk of thrombosis were increased (Tan et al., 2020). A fish oil-rich diet can improve endothelium-dependent vascular relaxation and delay the progression of atherosclerosis by upregulating the expression of MFN2 and OPA1 in aortas of ApoE^−/−^ mice fed with high-fat diet (Sun et al., 2014). In addition, the clinical study of Diaz-Morales et al. (Diaz-Morales et al., 2016) reported that compared with those in healthy volunteers, the mitochondrial fusion-related proteins MFN1/2 and OPA1 were significantly downregulated in circulating leukocytes isolated from diabetic patients. The decrease in leukocyte mitochondrial fusion and the increase in mitochondrial fission in diabetic patients were related to an increase in leukocyte-endothelial cell interaction, indicating that a higher degree of vascular inflammation is inseparable from the imbalance of endothelial cell mitochondrial dynamics.

### 4.2 Mitophagy and vascular pathology

Mitophagy is a type of organelle autophagy that prevents the accumulation of abnormal mitochondria (Jia et al., 2023). After mitochondrial degradation, amino acids and fatty acids are recycled to ensure the continuous growth and division of the existing mitochondrial network through mitochondrial biogenesis to meet the energy needs of cells (Wang L. et al., 2023). Mitochondrial autophagy is coordinated with mitochondrial biogenesis, ensuring a healthy mitochondrial network through the recycling of mitochondrial components (Wang et al., 2024).

Current researches have identified four initiation pathways for mitophagy: PTEN induced putative kinase 1 (PINK1)/Parkin pathway, BCL2/adenovirus E1B 19 kDa protein-interacting protein 3 (BNIP3)/NIP3-like protein X (NIX) pathway, the FUN14 domain containing 1 (FUNDC1) pathway, and the cardiolipin pathway. When mitophagy is initiated by the above-mentioned key proteins that initiate mitophagy, the autophagy-related protein microtubule-associated protein 1A/1B-light chain 3 (LC3) is processed into its cytoplasmic-localized LC3I, which is then combined with phosphatidylethanolamine (PE) on the phagosome and outer membrane to form LC3II (Chang et al., 2012; Zhu et al., 2022). LC3II binds to the mitophagy receptor protein, prompting the target mitochondria to be phagocytosed by phagosomes to form autophagosomes. Finally, lysosomes induce the hydrolysis and degradation of autophagosome proteins, nucleic acids and lipids, which are recovered by cells to restore balance.

Under pathological conditions, mitophagy is generally considered to be a protective mechanism. When damaged mitochondria cannot be repaired by mitochondrial fission or fusion, mitophagy is activated to remove damaged mitochondria and prevent the apoptosis caused by excessive mitochondrial damage. During atherosclerosis, excessive ROS production in macrophages has been shown to activate NLRP3 inflammasome formation and cause mitochondrial damage. Promoting Parkin-mediated mitophagy can reduce mitochondrial ROS production and NLRP3 inflammasome-induced mitochondrial damage and inhibit oxidative stress and the inflammatory response (Jin et al., 2022a). The inhibition of BNIP3-related mitophagy exacerbates oxidative stress-induced vascular injury and promotes osteoblast phenotype transformation and calcium deposition in vascular smooth muscle cells (Zhu et al., 2020). In the process of cardiac microvascular I/R, it was also found that the number of mitochondrial-lysosomal complexes in mouse cardiac microcirculation vascular ECs was significantly reduced, and the expression of Parkin was decreased, indicating that mitophagy was inhibited. The activation of mitophagy can protect ECs (Li et al., 2020a). However, some studies have shown that excessive activation of mitophagy under pathological conditions may play a role in promoting cell death. In a study of microcirculatory I/R, I/R activated Drp1-dependent mitochondrial fission, followed by upregulation of PINK1/Parkin, which was mediated by voltage-dependent anion channel 1 (VDAC1)/hexokinase 2 (HK2), and ultimately activated mitophagy-mediated cardiac thrombotic ECs death (Zhou et al., 2017b). An appropriate amount of mitophagy plays a protective role in the process of vascular pathology and helps to maintain the normal function and cell viability of vascular ECs. When mitophagy is overactivated, or still unable to correct mitochondrial damage, it has a synergistic trend with vascular endothelial damage.

### 4.3 Mitochondrial biogenesis and vascular pathology

Mitochondrial biogenesis is a dynamic process that stabilizes mitochondria to maintain their structure. Mitochondrial biogenesis is activated to ensure the function and quantity of normal cellular metabolism when the demand for cellular energy metabolism increases, or when increased activity or proliferation is required. Peroxisome proliferator-activated receptor coactivator (PGC-1α) is considered to be the main regulator of mitochondrial biogenesis, and can activate the expression of a variety of downstream transcription factors, including nuclear respiratory factors (NRF1/2), peroxisome proliferator-activated receptors (PPARs) and estrogen-related receptors (ERRs). NRF1/2 promotes the expression of nuclear-encoded mitochondrial transcription factor A (Tfam), which is responsible for the transcription of mtDNA (Gleyzer et al., 2005; Xiang D. et al., 2022). PPARs and ERRs are involved in the generation of nuclear proteins and control many aspects of mitochondrial oxidative metabolism, including fatty acid transport and oxidation, glucose utilization, the TCA cycle, and oxidative phosphorylation (OXPHOS) (Kim et al., 2021; Li Z. et al., 2021; McMeekin et al., 2021; Tian et al., 2023).

Under various pathological stimuli during ICM, mitochondrial biogenesis function is mainly impaired. Studies have shown that under constant high glucose induction, the level of ROS in ECs increases, the activation of PGC-1α is inhibited (Abdelzaher et al., 2016), and the expression levels of NRF1 and TFAM decrease (Xu et al., 2014). After exposure to cardiac microvascular I/R injury, the expression of PGC-1α and Tfam in mouse cardiac microcirculation vascular ECs is inhibited, and the function of mitochondrial biogenesis is impaired (Tan et al., 2020). In oxygen-glucose deprivation-mediated endothelial cell injury, promoting the activation of PGC-1α via therapeutic measures can upregulate the activity and expression of eNOS and improve vasodilation function (Wei et al., 2019). In addition, studies have shown that hypoxia/reoxygenation injury induces increased mitochondrial fission, decreased fusion, and impaired mitochondrial biogenesis in microvascular ECs. The activation of mitophagy can improve these conditions, upregulate SIRT3 and PGC-1α, and restore endothelial cell viability and proliferation (Wu et al., 2022). The results of this study reflect the mutual regulation between mitochondrial quality control links in the pathological process. Mitochondrial dynamics maintains the stability of mitochondrial morphology and function, while the cycle of mitophagy and mitochondrial biogenesis effectively promote the recovery of damaged mitochondria and the timely supplement of mitochondrial number. By regulating some of these links, ICM intervention and treatment can be achieved.

## 5 Targeted therapy of natural medicines

Studies have shown that many Chinese herbal medicines and their natural medicinal active ingredients have MQC and ER protection functions, which can resist oxidative stress, alleviate vascular inflammation, directly or indirectly improve vascular endothelial function and reduce vascular injury (Table 1).

**TABLE 1:** Effects of natural medicines on reducing vascular injury.

Category	Name	Classification	Mechanism	Experiment methods	References
Monomer	Taurine	Amino sulfonic acid	Inhibiting endoplasmic reticulum stress and reducing oxidative stress and apoptosis	*In vivo*; *in vitro*	Nonaka et al. (2001) Zulli et al. (2009)
Monomer	Physcion	Anthraquinone	Inhibiting oxidative stress and endoplasmic reticulum stress	*In vitro*	Wang et al. (2023d)
Monomer	Ursodeoxycholic Acid	Bile acid	Inhibiting endoplasmic reticulum stress and reducing oxidative stress and inflammatory response	*In vivo*; *in vitro*	Chung et al. (2015) Chung et al. (2016)
Monomer	Apocynin	Ketone	Inhibiting endoplasmic reticulum stress and reducing apoptosis	*In vitro*	Wu et al. (2018)
Monomer	Isorhapontigenin	Stilbenoid	Promoting mitochondrial fusion and inhibiting ferroptosis	*In vivo*; *in vitro*	Chen et al. (2023b)
Monomer	Diallyl trisulfide	Trisulfide	Inhibiting mitochondrial fission and cell apoptosis	*In vitro*	Hao et al. (2019)
Monomer	Mangiferin	Xanthone	Inhibiting endoplasmic reticulum stress and its related oxidative stress and inflammation	*In vitro*	Song et al. (2015)
Monomer	Berberine	Alkaloid	Promoting mitochondrial biogenesis against oxidative stress	*In vivo*	Arsenijevic et al. (2000) Wang et al. (2011)
Monomer	Tetramethylpyrazine	Alkaloid	Promoting mitochondrial biogenesis against oxidative stress Inhibiting endoplasmic reticulum stress	*In vitro*	Xu et al. (2014) Mak et al. (2017)
Monomer	Scutellarin	Flavonoid	Activating mitophagy against oxidative stress	*In vitro*	Xi et al. (2021)
Monomer	(−)-Epicatechin	Flavonoid	Activating mitochondrial biogenesis, enhancing metabolic capacity, and inhibiting oxidative stress	*In vitro*	Moreno-Ulloa et al. (2013) Ramírez-Sánchez et al. (2016) Keller et al. (2020)
Monomer	Naringenin	Flavonoid	Promoting mitochondrial biogenesis	*In vivo*	Wang et al. (2023a)
Monomer	Icariin	Flavonoid	Enhancing mitophagy to inhibit ferroptosis Inhibiting endoplasmic reticulum stress and reducing oxidative stress injury	*In vivo*; *in vitro*	Wang et al. (2019a) Yao et al. (2021) Wang et al. (2023c)
Monomer	Quercetin	Flavonoid	Reducing excessive mitochondrial fission Relieving endoplasmic reticulum stress, and reducing subsequent inflammatory response and apoptosis	*In vivo*; *in vitro*	Suganya et al. (2014) Wu et al. (2014a) Cai et al. (2017) Chen et al. (2019)
Monomer	Luteolin	Flavonoid	Resisting oxidative stress and endoplasmic reticulum stress, and inhibiting subsequent inflammatory response and apoptosis	*In vitro*	Wu et al. (2014a)
Monomer	Epigallocatechin gallate	Flavonoid	Resisting oxidative stress and endoplasmic reticulum stress, and inhibiting subsequent inflammatory response and apoptosis	*In vitro*	Wu et al. (2014a)
Monomer	Ferulic acid	Phenol	Activating mitochondrial biogenesis, mitochondrial fusion and fission, and inhibiting oxidative stress and cell apoptosis	*In vivo*; clinical trial	Perez-Ternero et al. (2017)
Monomer	Vanillic acid	Phenol	Promoting mitochondrial biogenesis against oxidative stress	*In vitro*	Ma et al. (2019)
Monomer	Paeonol	Phenol	Inhibiting endoplasmic reticulum stress and reducing oxidative stress	*In vivo*; *in vitro*	Choy et al. (2016) Choy et al. (2017)
Monomer	Salidroside	Phenolic glycoside	Activating mitochondrial biogenesis Inhibiting endoplasmic reticulum stress	*In vitro*	Xing et al. (2014) Zhu et al. (2017)
Monomer	Chlorogenic acid	Polyphenol	Promoting mitochondrial biogenesis against oxidative stress	*In vitro*	Tsai et al. (2018)
Monomer	Caffeic acid	Polyphenol	Inhibiting oxidative stress and endoplasmic reticulum stress, and reducing the inflammatory response	*In vitro*	Toma et al. (2017)
Monomer	Hydroxytyrosol	Polyphenol	Promoting mitochondrial biogenesis	*In vitro*	Calabriso et al. (2018)
Monomer	Punicalagin	Polyphenol	Promoting mitochondrial biogenesis	*In vitro*	Liu et al. (2019)
Monomer	Piceatannol	Polyphenol	Inhibiting oxidative stress, endoplasmic reticulum stress, and apoptosis	*In vitro*	Kil et al. (2017)
Monomer	Polydatin	Polyphenol	Enhancing mitochondrial fission, inhibiting oxidative stress and cell pyroptosis	*In vitro*	Shah et al. (2023)
Monomer	Resveratrol	Polyphenol	Promoting mitochondrial biogenesis, mitochondrial fusion, and mitophagy	*In vivo*; *in vitro*	Csiszar et al. (2009), Davinelli et al. (2013) Yang et al. (2019) Li et al. (2020b)
Monomer	Rosolic acid	Polyphenol	Inhibiting oxidative stress and endoplasmic reticulum stress	*In vitro*	Foresti et al. (2005)
Monomer	Salvianolic acid B	Polyphenol	Inhibiting mitochondrial fission, mitophagy, and apoptosis Relieving endoplasmic reticulum stress against oxidative stress and pyroptosis	*In vivo*; *in vitro*	Ko et al. (2020) Xiang et al. (2022b) Tang et al. (2022)
Monomer	Astragaloside IV	Saponin	Inhibiting oxidative stress-induced endoplasmic reticulum stress, and reducing subsequent inflammatory response and apoptosis	*In vitro*	Zhao et al. (2015)
Monomer	Ginsenoside Rb2	Saponin	Reducing inflammation and endoplasmic reticulum stress, and inhibiting apoptosis and adhesion of THP-1 monocytes to HUVECs	*In vitro*	Sun et al. (2020)
Monomer	Ginsenoside Rh1	Saponin	Inhibiting oxidative stress, endoplasmic reticulum stress, and apoptosis	*In vivo*; *in vitro*	Jin et al. (2022b)
Monomer	Ginsentide TP1	Saponin	Inhibiting endoplasmic reticulum stress and oxidative stress	*In vitro*	Dutta et al. (2023)
Monomer	Paeoniflorin	Terpenoid	Inhibiting endoplasmic reticulum stress and reducing inflammatory response	*In vitro*	Chen et al. (2018)
Monomer	Triptolide	Diterpenoid	Activating mitochondrial biogenesis to inhibit endoplasmic reticulum stress and apoptosis	*In vitro*	Xiang et al. (2020)
Monomer	Corosolic acid	Triterpenoid	Inhibiting mitochondrial fission and oxidative stress	*In vivo*; *in vitro*	Li et al. (2016)
Monomer	Cycloastragenol	Triterpenoid	Inhibiting oxidative stress-induced endoplasmic reticulum stress, and reducing subsequent inflammatory response and apoptosis	*In vitro*	Zhao et al. (2015)
Monomer	Ilexgenin A	Triterpenoid	Promoting mitochondrial biogenesis to inhibit mitochondrial fission and oxidative stress Reducing endoplasmic reticulum stress and inflammatory activation	*In vitro*	Zhu et al. (2019) Li et al. (2015)
Herb	Ethanol extract of propolis	—	Inhibiting endoplasmic reticulum stress and reducing apoptosis	*In vitro*	Tian et al. (2015)
Formula	*Buyang huanwu* decoction	—	Inhibiting mitochondrial fission and oxidative stress	*In vivo*; *in vitro*	Tong et al. (2023)
Formula	*Shuangshen ningxin* formula	—	Inhibiting mitochondrial fission	*In vivo*; *in vitro*	Liu et al. (2024b)
Formula	*Ginseng-Sanqi-Chuanxiong (GSC)* extracts	—	Activating mitophagy against oxidative stress	*In vitro*	Wang et al. (2020)
Formula	*Shenlian* extract	—	Inhibiting excessive mitophagy	*In vivo*; *in vitro*	Li et al. (2023a)
Formula	*Yi Mai* granule	—	Promoting mitophagy to reduce inflammatory response	*In vivo*; *in vitro*	Kong et al. (2024)
Formula	*Dangua Fang*	—	Activating mitochondrial biogenesis, enhancing metabolic capacity, and inhibiting oxidative stress	*In vitro*	Xianpei et al. (2022)
Formula	*Guanxinkang* decoction	—	Inhibiting endoplasmic reticulum stress against apoptosis	*In vitro*	Wang et al. (2014)
Formula	*Danzhi jiangtang* capsule	—	Reducing oxidative stress, endoplasmic reticulum stress, and inflammatory response	*In vivo*; *in vitro*	Lu et al. (2018)

### 5.1 Mitochondrial quality control protection function

The mechanisms involved in mitochondrial dynamics, mitophagy and mitochondrial biogenesis mentioned above are all possible targets for natural drugs to improve mitochondrial quality control disorders during ICM.

For mitochondrial dynamics, natural drugs mainly inhibit excessive mitochondrial fission in pathological conditions by regulating the expression of DRP1 and FIS1 or promote mitochondrial fusion by regulating the expression of MFN1, MFN2 and OPA1, thereby exerting a protective effect on mitochondria and cells. Corosolic acid is a natural triterpene with antioxidant activity. In ECs, it activates AMPK in a IKB1-dependent manner, induces phosphorylation of the Ser637 site of DRP1, thereby inhibiting mitochondrial fission and resisting subsequent oxidative stress. Oral administration of corosolic acid in high-fat diet mice can replicate similar regulation in aortic endothelium (Li et al., 2016). Diallyl trisulfide is an organic polysulfide found in *Allium sativum* L. Studies have shown that Diallyl trisulfide can inhibit high glucose-induced HUVECs apoptosis in an AMPK-dependent manner by inhibiting DRP1-mediated mitochondrial fission (Hao et al., 2019). Isorhapontigenin is extracted from *Gnetum cleistostachyum* C.Y.Cheng, and has been proved to have good anti-inflammatory and anti-oxidative stress effects in many studies. Chen Y. et al., 2023) pointed out that isorhapontigenin promoted mitochondrial fusion through peroxiredoxin 2 (PRDX2)/MFN2, inhibited ACSL4-mediated ferroptosis of mitochondria-associated ECs and improved microvascular density and perfusion in db/db mice. Polydatin, which is extracted from *Polygonum cuspidatum* Sieb. et Zucc., enhances DRP1-mediated mitochondrial fission, reduces ROS production, and improves pyroptosis of HUVECs and the aorta in diabetic rats through the NLRP3/Caspase1/IL-1β pathway (Shah et al., 2023).

*Buyang huanwu* decoction (BYHWD, a TCM prescription) has been shown to reduce mitochondrial fission (decreased expression levels of DRP1 and FIS1) through AMPK activation, reduce ROS production, and improve NO production in diabetic ApoE mouse models and HUVECs exposed to high glucose (Tong et al., 2023). *Shuangshen ningxin* formula, another TCM prescription, has also been shown to reduce mitochondrial fission by inhibiting the nuclear receptor subfamily 4 group A member 1 (NR4A1)/MFF/Drp1 pathway *in vivo* and *in vitro* experiments, and plays a protective role in cardiac microvessels (Liu Z. et al., 2024).

Owing to the “double-sided” characteristics of mitophagy, either overexcitation or inhibition of mitophagy may be responsible for the pathological state. Thus, there are two trends in the action of natural drugs. Scutellarin is one of the active components of *Scutellariae Radix*. It can upregulate mitophagy through the PINK1/Parkin signalling pathway, fight against excessive ROS production, and protect vascular ECs from hyperglycemia-induced damage (Xi et al., 2021). Ginseng-Sanqi-Chuanxiong (GSC) extracts, a composite extract of *Ginseng Radix Et Rhizoma*, *Notoginseng Radix Et Rhizoma*, and *Chuanxiong Rhizoma*, was shown to activate mitophagy via the AMPK pathway, and was able to reduce the significant increase in mitochondrial ROS levels in HAECs under high glucose and palmitate stress conditions (Wang et al., 2020). Shenlian extract is a combination of *Salviae miltiorrhizae Radix et Rhizoma* and *Andrographis Herba*. *In vivo* and *in vitro* experiments have shown that Shenlian extract can regulate mitochondrial dysfunction and protect microvascular function by inhibiting mitophagy via the PINK/Parkin pathway (Li J. et al., 2023). *Yi Mai* granule is a TCM combination preparation, which regulates proinflammatory factors and blood lipid levels by promoting PINK1/MFN2/Parkin-mediated mitophagy, plays an endothelial protective role *in vivo* and *in vitro* experiments (Kong et al., 2024).

In terms of mitochondrial biogenesis, the mitochondrial biogenesis factors PGC-1α, Nrf-1, and Tfam are the main intervention targets of natural drugs and their monomers. Improvements in mitochondrial quality, mtDNA content, ATP production, oxidative phosphorylation function, and antioxidant capacity are the main follow-up effects. In the fight against ICM inflammatory vascular injury, improving mitochondrial biogenesis seems to be one of the most important ways for natural drugs to exert their efficacy.

As the main active ingredient of *Coptidis Rhizoma*, berberine has been shown to promote mitochondrial biogenesis in an AMPK-dependent manner in ApoE^−/−^ mice, increase the level of the mitochondrial-derived ROS regulator uncoupling protein 2 (UCP2), and resist oxidative stress (Arsenijevic et al., 2000; Wang et al., 2011). (−)-Epicatechin is a natural flavanol compound that enhances citrate synthase activity in bovine coronary artery ECs, increases the levels of oxidatively phosphorylated proteins (complexes I and II), activates mitochondrial biogenesis, and stimulates mitochondrial function (Moreno-Ulloa et al., 2013). In addition, (−)-epicatechinis ameliorated the hyperglycemia-induced decrease in eNOS activity in HCAECs by activating mitochondrial biogenesis pathway (Ramírez-Sánchez et al., 2016), and was found to regulate the activity of mitochondrial complexes and reduce high glucose-mediated increasing ROS levels in HUVECs (Keller et al., 2020).

Chlorogenic acid, hydroxytyrosol and punicalagin are all natural polyphenolic compounds that are widely found in a variety of natural plant medicines. Chlorogenic acid pretreatment can increase the activity level of SIRT1 deacetylase, reverse the SIRT1/AMPK/PGC-1 activity damaged by ox-LDL, and reduce the degree of oxidative stress in HUVECs (Tsai et al., 2018). Hydroxytyrosol has been shown to promote mitochondrial biogenesis, increase mtDNA content, and attenuate endothelial dysfunction and pathological angiogenesis (Calabriso et al., 2018). Punicalagin, which is extracted from *Punica granatum* L., can promote mitochondrial biogenesis through the forkhead box O1 (FOXO1) pathway and improve hyperlipidemia-induced endothelial dysfunction (Liu et al., 2019). Vanillic acid is a phenolic acid compound widely distributed in natural plants. In palmitic acid-stimulated HUVECs, vanillic acid promoted the expression of p-Nrf2, HO-1, SIRT1, and PGC-1α through the LKB1/AMPK signalling pathway, and reduced the levels of ROS and malondialdehyde (MDA) to alleviate oxidative stress (Ma et al., 2019). Naringenin is a natural flavonoid widely distributed in plants. Its treatment can increase the expression of genes related to mitochondrial biogenesis (SIRT1, FOXO3a and PGC1α) in the aorta of ApoE^−/−^ mice and improve vascular aging and atherosclerosis (Wang J. et al., 2023).

*Dangua Fang,* a TCM compound, can increase the expression of PGC-1α in ECs, regulate mitochondrial respiratory chain function, restore the mitochondrial membrane potential (MMP), and resist oxidative stress-induced endothelial cell injury (Xianpei et al., 2022).

In addition, the effects of some natural medicines on various aspects of MQC mechanisms have been comprehensively studied.

Resveratrol is a natural polyphenolic compound found in a variety of plants such as *Polygoni Cuspidati Rhizoma et Radix* and *Vitis*. *In vitro* studies revealed that resveratrol was able to increase the mitochondrial mass and mtDNA content and regulate the bioavailability of NO by inducing the upregulation of mitochondrial biogenesis factors (PGC-1α, Nrf-1, and Tfam) in HCAECs. Long-term resveratrol treatment corrects damage to mitochondrial biogenesis in the aorta of type 2 diabetic mice (Csiszar et al., 2009). Similarly, in HUVECs, the co-administration of resveratrol and equol, a natural flavonoid compound, induced the expression of the mitochondrial biogenesis factors PGC1-α, Tfam, and Nrf-1 via SIRT1, and increased mitochondrial mass and mitochondrial DNA content (Davinelli et al., 2013). In addition, resveratrol increased the protein levels of MFN1, MFN2 and OPA1, promoted mitochondrial fusion, and resisted palmitic acid-mediated oxidative damage to ECs (Yang et al., 2019). It can upregulate BNIP3-related mitophagy through HIF1/AMPK signaling pathway and prevent ox-LDL-mediated mitochondrial respiratory complex inactivation to promote endothelial cell survival (Li et al., 2020b).

Ferulic acid is the main active ingredient of *Chuanxiong Rhizoma.* It can inhibit the downregulation of vascular endothelial mitochondrial biogenesis markers (PGC-1α, PGC-1β, and NRF-1) and the decrease in fusion (Mfn1and Mfn2 levels decreased) and fission (FIS1 levels decreased) in ApoE^−/−^ mice induced by a high-fat diet, thereby inhibiting the development of aortic atherosclerotic plaques and oxidative stress in mice. Clinical studies have shown that the intake of ferulic acid in healthy volunteers can reduce the activity of NADPH oxidase, superoxide release and apoptosis of peripheral blood monocytes, and improve the differentiation and proliferation of endothelial progenitor cells (Perez-Ternero et al., 2017).

### 5.2 ER protection function

The TCM plays an ER protection role mainly by resisting ERS. It can also inhibit the upstream and downstream oxidative stress of ERS and subsequently mediate the inflammatory response and programmed cell death and reduce vascular injury. Its ER protection function is reflected mainly in the regulation of ERS markers.

Taurine is one of the active components of *Bovis Calculus,* a natural Chinese medicine derived from animals, and is also found in a variety of animal foods. Studies have shown that taurine can improve homocysteine-induced vascular smooth muscle cells (VSMCs) ERS, downregulate the expression of GRP78 mRNA, inhibit PERK, and restore extracellular superoxide dismutase (EC-SOD) secretion to reduce oxidative stresss (Nonaka et al., 2001). In *in vivo* experiments, dietary taurine supplementation can reduce the expression of CHOP protein in ECs, inhibit the ERS-mediated apoptosis of ECs, and reduce the atherosclerosis of left main coronary artery in rabbits (Zulli et al., 2009). Ursodeoxycholic acid (UDCA) is a widely used drug and the main active ingredient of the precious traditional animal medicine *Fel Ursi*. A study by Chung et al. (2015), Chung et al. (2016) revealed out that UCDA blocked ERS and its downstream signalling pathways (p-PERK, XBP1, ATF6 and CHOP) in high glucose-treated ECs, inhibited subsequent oxidative stress and pro-inflammatory responses (ROS production and NF-κB activation), and inhibited the formation of atherosclerotic plaques. In *in vivo* experiments, UCDA has been shown to exert anti-atherosclerotic activity by inhibiting ERS in atherosclerotic mouse models caused by blood flow disorders and diabetic mouse models. Propolis is a colloidal substance formed by mixing plant resin collected by bees with secretions such as maxillary glands and wax glands and a small amount of pollen. It has a long history of application in traditional medicine. A study by Hua et al. (Tian et al., 2015) showed that ethanol extract of propolis inhibited ERS/CHOP pathway-mediated apoptosis by reducing the expression of PERK, eIF2α, GRP78 and CHOP, and played a protective role in ox-LDL-induced macrophage toxicity.

Apocynin, which is isolated from *Picrorhiza Kurroa*, is widely used as an inhibitor of NADPH oxidase (NOX). Studies have shown that Apocynin can participate in protecting ECs from ERS-induced apoptosis by promoting the expression of IRE1α at the mRNA and protein levels (Wu et al., 2018). Knockdown of IRE1α relieved this protective effect.

Astragaloside IV and cycloastragenol are the main active components of *Astragali Radix*. Zhao et al. (2015) reported that both of them could inhibit the production of ROS, the phosphorylation of IRE1α and the subsequent activation of TXNIP/NLRP3, inhibit ROS/ERS/inflammation, and play an anti-apoptotic role in palmitic acid-stimulated ECs.

Mangiferin is the active ingredient of traditional medicine *Anemarrhenae Rhizoma* and *Belamcandae Rhizoma*. In high glucose-treated ECs, mangiferin can effectively inhibit ERS and ERS-related oxidative stress and inflammation. The specific mechanism involves reducing reduce the phosphorylation of IRE1α through the AMPK pathway, inhibiting the expression of TXNIP and NLRP3, and reducing the production of ROS, IL-1β and IL-6 (Song et al., 2015).

Paeoniflorin is the main active ingredient of *Paeoniae Radix Alba*. Through the IRE1α/NF-κb pathway, paeoniflorin inhibited the overexpression of ERS markers (GRP78 and CHOP) and inflammatory cytokines (IL-6 and MCP-1) and reduced lipopolysaccharide (LPS) -induced HUVECs injury (Chen et al., 2018).

Paeonolis is present in a variety of plant medicines and is the main active ingredient of *Moutan Cortex*. It can inhibit the protein expression of ERS markers (eIF2α, ATF6, and GRP78) in the vascular wall through the AMPK/PPARδ signalling pathway, reduce ROS production, ameliorate the damage of aortic endothelium-dependent relaxation in mice, and increase the bioavailability of NO (Choy et al., 2016; Choy et al., 2017).

Luteolin and epigallocatechin gallate (EGCG) are natural flavonoids. EGCG is present mainly in *Camellia sinensis* (L.) Kuntze, and luteolin can be found in a variety of plant medicines. Both of them can reduce the production of ROS and the activation of TXNIP through the activation of AMPK signalling pathway, which means that they play a role in resisting oxidative stress and ERS. They can also inhibit the subsequent expression of the NLRP3 inflammasome and IL-1β, and protect HUVECs from apoptosis by restoring MMP and inhibiting caspase-3 activity (Wu J. et al., 2014).

Caffeic acid is a phenolic acid widely found in many plants. By inhibiting receptor for advanced glycation endproducts (RAGE), NOX4-dependent oxidative stress, and ERS (promoting GRP78 protein expression, reducing EIF2α phosphorylation and CHOP expression), caffeic acid can reduce the secretion of CRP, VCAM-1 and MCP-1 in human ECs treated with glycosylated low-density lipoprotein (g-LDL) (Toma et al., 2017).

*Ginseng Radix Et Rhizoma* is the dried root and rhizome of *Panax ginseng* C. A. Mey., which is a valuable Chinese herbal medicine containing a large number of saponins (Yu et al., 2020). Under LPS conditions, ginsenoside Rb2 can reduce inflammation and ERS in HUVECs and THP-1 monocytes, thereby inhibiting the apoptosis and adhesion of THP-1 monocytes to HUVECs (Sun et al., 2020). Ginsenoside Rh1 has been shown to reduce LPS-induced ECs inflammation and apoptosis by blocking the binding of LPS to TLR2 and TLR4, thus inhibiting STAT3/NF-κB inflammatory pathway and PERK/CHOP/ERO1-α ERS signaling pathway. In the *in vivo* model, ginsenoside Rh1 can also effectively protect the ER and rescue the tight junctions of ECs damaged by LPS (Jin et al., 2022b). Ginsentide TP1 is a recently discovered heat-stable microprotein in *Ginseng Radix Et Rhizoma*. It has been shown to protect HUVECs from hypoxia and ERS, restore NO signal transduction and bioavailability, and reduce oxidative stress (Dutta et al., 2023).

*Guanxinkang* decoction and *Danzhi jiangtang* capsule are both TCM prescriptions. Studies have shown that *Guanxinkang* decoctioncan can inhibit endoplasmic reticulum stress (reduce GRP78 expression), increase the MMP, and reverse homocysteine-mediated HUVECs apoptosis. In addition, its antagonistic effect increased with the increase of concentration and action time (Wang et al., 2014). In high-fat diet-fed rats and palmitic acid-treated HUVECs, *Danzhi jiangtang* capsule can effectively reduce oxidative stress and ER stress (decreased expression of IRE1α, XBP1, CHOP and GRP78), and reduce lipid deposition and the inflammatory response (Lu et al., 2018).

### 5.3 The regulatory function of the interaction mechanism

Previous studies have shown that some natural drugs can play regulatory roles in both MQC and ER function.

Salidroside is the main active ingredient of the natural medicine *Rhodiolae Crenulatae Radix et Rhizoma*. Salidroside pretreatment of HUVECs significantly upregulated PGC-1 and Tfam, promoted mitochondrial biogenesis, and improved endothelial cell mitochondrial quality and ATP production. The toxicity of H_2_O_2_ to cells was reduced, and endothelium-dependent vascular relaxation was restored (Xing et al., 2014). In addition, salidroside can inhibit homocysteine-induced activation of Bip and CHOP, as well as phosphorylation of PERK and IRE1α, and regulate ERS to exert a protective effect on HUVECs (Zhu et al., 2017).

Tetramethylpyrazine is one of the active components of the natural medicine *Chuanxiong Rhizoma*. It can reverse the inhibition of sirtuin 1 (SIRT1), PGC-1α, Nrf-1, and Tfam in ECs caused by high glucose and play an antioxidant role (Xu et al., 2014). In addition, studies by Mak et al. (2017) showed that tetramethylpyrazine alleviated endothelium-dependent vasodilatation injury caused by angiotensin-II (Ang-II) by inhibiting the expression of the ERS-related markers GRP78, ATF6, pPERK and p-IRE1 in porcine coronary ECs.

Ilexgenin A, which is extracted from *Ilex hainanensis* Merr., activated NRF2 expression by promoting mitochondrial biogenesis and inhibited palmitate-induced excessive mitochondrial fission (increasing DRP1 expression level) and oxidative stress (Zhu et al., 2019). In addition, Ilexgenin A can attenuate IRE1 and PERK phosphorylation by regulating AMPK, inhibit palmitate-induced ERS and subsequent TXNIP/NLRP3 inflammasome activation, and improve endothelial dysfunction (Li et al., 2015).

Triptolide is a diterpene lactone epoxide compound extracted from *Tripterygium wilfordii* Hook. f. that can regulate the activation of the PPARs/PGC-1α pathway, play a role in ERS (downregulation of GRP78, XBP1, CHOP levels) and apoptosis (downregulation of Bax and caspase-3, and upregulation of Bcl-2 levels) of HUVECs (Xiang et al., 2020).

Icariin is the main active ingredient of *Epimedii Folium*, which can enhance mitophagy by promoting autophagosome-lysosome fusion and inhibit ferroptosis induced by ox-LDL (Wang X. et al., 2023). In addition, icariin exerted a dose-dependent protective effect on H_2_O_2_-induced vascular ECs injury by inhibiting ERS (reducing GRP78, ATF4 and eIF2α protein expression), enhancing SOD and glutathione peroxidase (GSH-Px) activities, and alleviating oxidative stress injury (Wang F. et al., 2019). *In vivo and in vitro* studies have shown that the protective effect of icariin on ER is related to the activation of PPARα/Sirt1/AMPKα pathway (Yao et al., 2021).

Salvianolic acid B is the main active ingredient of traditional natural medicine *Salviae miltiorrhizae Radix et Rhizoma*, which has a good protective effect on MQC and ER function. Studies have shown that salvianolic acid B can inhibit the increase in p-DRP1 and FIS1 levels induced by oxLDL and high glucose, which means that it can inhibit the mitochondrial fission of ECs (Ko et al., 2020). In the thoracic aorta of diabetic mice and HUVECs induced by HG-carbonyl cyanide m-chlorophenyl hydrazone (CCCP), Salvianolic acid B can significantly increase the expression of Bcl-2, reduce the expression of BAX, Beclin1, Parkin and Pink1, which protected ECs from mitophagy and apoptosis (Xiang J. et al., 2022). In addition, by regulating the AMPK/FoxO4/KLF2 and Syndecan-4/Ras-related C3 botulinum toxin substrate 1 (Rac1)/ATF2 pathways, salvianolic acid B can alleviate ERS and oxidative stress, and inhibit NLRP3 inflammasome-mediated ECs pyroptosis (Tang et al., 2022).

Quercetin is a flavonoid widely found in a variety of natural plant medicines. It can reduce mitochondrial fragmentation and restore vascular endothelial insulin sensitivity by inhibiting Drp1 levels and serine 616 phosphorylation in the ECs of obese mice (Chen et al., 2019). In addition, quercetin was shown to reduce the expression levels of GRP78 and CHOP to inhibit ERS in HUVECs, reduce the expression of the NLRP3 inflammasome and IL-1β and inhibit apoptosis. AMPK signaling pathway may be involved in mediating this effect (Suganya et al., 2014; Wu J. et al., 2014). For HUVECs treated with high glucose, quercetin also played a role in inhibiting ERS and the subsequent inflammatory response (Cai et al., 2017).

In general, the regulatory mechanisms of some natural drugs on both mitochondria and ER have been relatively well studied. However, further research is necessary to determine whether there are upstream and downstream correlations between the two regulatory mechanisms and whether there is a common upstream regulatory pathway.

Additionally, the crosstalk between oxidative stress and ERS intervened by TCM has been widely studied in recent years. Among these, Nrf2 is a crucial target for natural drugs to regulate both oxidative stress and ERS. *Rhei Radix et Rhizoma*, derived from the dried roots and rhizomes of *Rheum palmatum* L., is a traditional Chinese herbal medicine with a long history of application. Piceatannol is its active ingredient. Studies have shown that piceatannol pretreatment of ECs can activate Nrf2/heme oxygenase-1 (HO-1) expression to reduce GRP78 and CHOP expression and XBP1 mRNA splicing. Sulfur-containing amino acid homocysteine-induced oxidative stress, ERS and apoptosis were therefore inhibited (Kil et al., 2017). Similarly, as one of the active components of *Rhei Radix et Rhizoma*, physcion can increase the activation of eNOS/Nrf2 signalling, inhibit palmitic acid-induced oxidative stress and ERS in HUVECs (inhibit the expression of GRP78 and its downstream proteins PERK, p-EIF2α, ATF4, and CHOP), and prevent the damage to endothelium-dependent relaxation (Wang Y.-H. et al., 2023). Rosolic acid is an important polyphenol extracted from *Plantago asiatica* L.with good cardiovascular benefits (Foresti et al., 2005). Naresh Amin et al. (2021) pointed out that rosolic acid can increase the activity of antioxidant enzymes SOD, catalase (CAT) and GSH-Px by activating Nrf2, restore redox homeostasis, weaken endoplasmic reticulum stress in ECs, and exert endothelial protection.

## 6 Conclusion

ICM has always been a clinical concern and research challenge. Despite significant advances in the treatment of myocardial ischemic injury, coronary vascular injury, which is a key pathological change in the progression of ICM, remains to be focused on. In this process, inflammation and oxidative stress interact with each other and trigger cell death, which is responsible for coronary microvascular injury. Imbalances in ER function and MQC are important potential factors for inflammation and oxidative stress.

However, current research on coronary artery injury focuses on the early stages of ICM, such as atherosclerosis. The research objects are mainly ECs treated with high glucose, palmitic acid, and homocysteine, or animal models of diabetes and atherosclerosis, with fewer explorations of vascular injury in the later stages of ICM. Therefore, it is necessary to pay more attention to the study of coronary microvascular or microcirculation injury in subjects more closely related to the later stage of ICM, such as vascular ECs and vascular smooth muscle cells treated with glucose/oxygen deprivation or hypoxia/reoxygenation and animal models of cardiac microcirculation I/R or myocardial infarction. This can provide a comprehensive understanding of the role of inflammation and oxidative stress in ICM.

Currently, although many studies have identified markers of ER or mitochondrial dysfunction, there are no specific biomarkers that can target early detection of ICM-related vascular injury. Therefore, the identification of specific ER or mitochondrial dysfunction biomarkers closely related to ICM vascular injury is critical for a comprehensive study of this process.

In addition, owing to the wide variety of TCMs and their the complex chemical composition, although many basic studies have focused on the targeted regulation of vascular injury by natural drugs, few drugs have really entered the clinical research stage. Therefore, it is necessary to focus on a specific prescription, drug, or natural monomer, and actively carry out clinical research. This can accelerate the process of drug realization from basic research to clinical application, and realize the translation of findings on herbal medicines into standardized treatment protocols for ICM.

According to this review, among the TCM monomers that have been reported to improve vascular injury, flavonoids and polyphenols account for a large proportion. They have good intervention effects on oxidative stress and ERS, and are widely found in plant-derived TCMs. Therefore, it is possible to consider the screening and in-depth study of natural plant medicines rich in flavonoids and polyphenols. These findings provide fast and valuable ideas for the screening of effective monomer drugs and the development of new drugs against ICM. Finally, ICM patients must follow their doctor’s guidance for long-term use of herbal therapies and have regular follow-up visits to evaluate the efficacy and determine the appropriate treatment
